# Fabrication of Chitosan/Polypyrrole‐coated poly(L‐lactic acid)/Polycaprolactone aligned fibre films for enhancement of neural cell compatibility and neurite growth

**DOI:** 10.1111/cpr.12588

**Published:** 2019-04-11

**Authors:** Yaxuan Xu, Zhongbing Huang, Ximing Pu, Guangfu Yin, Jiankai Zhang

**Affiliations:** ^1^ College of Materials Science and Engineering Sichuan University Chengdu China

**Keywords:** cell differentiation, chitosan, conductive aligned fibre‐film, electrical stimulation, neurite growth

## Abstract

**Objective:**

Chitosan (CS) and polycaprolactone (PCL) were added into a nerve scaffold of poly(L‐lactide acid) (PLLA)/polypyrrole (PPy)‐based fibre films to solve the unmatch with the nerve strength and the aseptic inflammation from PLLA.

**Methods:**

Poly (L‐lactide acid)‐polycaprolactone (PLLA/PCL) fibre films coated with chitosan (CS) and polypyrrole (PPy) were prepared by electrospinning of aligned PLLA/PCL fibres, electrochemical deposition of PPy nanoparticles and in situ doping of CS in PPy. PC12 cells were electrically stimulated with 100 mV for 2 hours every day via CS/PPy‐PLLA/PCL fibre film to promote the neurite growth.

**Results:**

The surface conductivity and tensile strength of CS/PPy‐PLLA/PCL fibre films were 1.03 s/m and 13 MPa, respectively. CS content in fibre films was about 7.5 mg/cm^2^, improving the pH value (reached to 5.1) of immersion solution of the fibre film at 16 days. Compared with PPy‐PLLA/PCL fibre film, more and longer axons were grown out from PC12 cells cultured on CS/PPy‐PLLA/PCL fibre film, indicating the positive effect of CS in fibre film on axon growth. The cell differentiation rate and neurite length on CS/PPy‐PLLA/PCL fibre film reached to 38% and 75 μm, respectively. These results suggest the promotion of electrical stimulation on neurite growth and alignment.

**Conclusions:**

A synergistic mechanism about the promotion of CS, electrical stimulation and aligned fibres on PC12 cells differentiation, axon outgrowth was proposed. These results indicated the potential application of CS/PPy‐PLLA/PCL fibre film in the field of the nerve repair and regeneration.

## 1 INTRODUCTION

For nerve cell adhesion, migration and subsequent tissue regeneration, supporting materials have been widely applied in tissue engineering (TE). These materials include natural macromolecules, synthetic polymers and their composite materials.^1^, ^2^, ^3^ Due to the particularity of nerve plerosis, the repair scaffold material should possess several properties as follows: good biocompatibility, aligned structure for the guidance of axon growth,^4^ electrical conductivity for the stimulation of axon growth,^5^ appropriate mechanical property,^6^ biodegradability for avoiding two times the surgery and aseptic inflammation.^7^

Recently, some reported materials have met above two‐three targets. For instance, poly(L‐lactic acid) (PLLA) conduits presented the better results of nerve regeneration in the rat sciatic nerve model.^8^ Yang et al reported that neuronal stem cells could grow along the aligned direction of PLLA fibre.^9^ However, these aligned PLLA fibres did not possess electrical conductivity and could not electrically stimulate the axon growth. The application of conductive polymers in the peripheral nerve repair was widely investigated, due to their good bio‐compatibility and the ability of transmitting electrical signal to the contacted tissue.^10^, ^11^, ^12^, ^13^ Polypyrrole (PPy), as a commonly used conductive polymer with good biocompatibility, has been investigated for the application in nerve repair and regeneration.^14^ Rivers et al reported that electrical stimulation (ES) through PPy film could accelerate neurite outgrowth.^15^ George et al prepared a PPy tube, which could be placed in transected rat sciatic nerves and supported nerve regeneration for over 8 weeks.^16^ However,this conduit could not guide axon growth during the nerve repair. These reports suggested that the single material could not meet all requirements of the nerve repair. Therefore, composite materials were prepared for nerve plerosis. Zhou et al cultured PC12 cells (a cell line derived from a pheochromocytoma of the rat adrenal medulla with an embryonic origin from the neural crest)^17^ on conductive PPy/PLLA fibre films, showing good results of neurite adhesion, alignment and elongation.^18^ Some reports also indicated that PPy‐coated fibre films could be applied in neural tissue engineering, due to their biocompatibility, degradation, aligned structure and electrical conductivity.^19^, ^20^

As a degradable scaffold, PLLA was easy to be electrospun into fibre films with unique regular topography.^21^, ^22^ However, its ductility was lower than normal nerve tissue.^23^ Moreover, the lactic acids from PLLA degradation in the implanted site maybe induce aseptic inflammation.^7^, ^22^ These results indicated that PLLA fibre films could not completely meet the requirements of nerve repair. Therefore, PLLA fibre film need to be integrated with other materials to improve their combination property, such as polycaprolactone (PCL) and Chitosan (CS).

Some studies demonstrated that PLLA/PCL scaffold prepared via electrospinning could allow cells adhesion and proliferation.^24^, ^25^ Moreover, immune‐histochemical examination showed that PLLA/PCL fibres provoked only a modest inflammatory response as nerve repair material.^26^ Due to its toughness,^27^ PCL was mixed with PLLA to toughen PLLA scaffold and did not decrease the biodegradability of the scaffold.^28^ In addition, the report of Cooper et al indicated that aligned PCL/CS fibre films could be also used in tissue engineering.^29^

Chitosan has gradually become well‐accepted biomaterial for recent years, due to its good biocompatibility and biodegradability.^30^, ^31^ Previous studies demonstrated that CS was neural cyto‐compatible.^32^ Moreover, CS coating could promote the growth of the dorsal root ganglion cell on scaffold material.^33^ Zhao et al reported a study on the degradation products of CS, chitooligosaccharides, which could promote nerve repair.^34^ Furthermore, CS could improve neural cell adhesion under weak acidic condition,^29^, ^35^ because the products of CS degradation were weakly alkal.^36^

In this work, a conductive CS/PPy‐coated PLLA/PCL fibre scaffold was prepared, in order to improve the mechanical matching of PLLA fibres with nerve tissue and the micro‐environment of neurite growth in PLLA‐based scaffolds via PCL addition and the in situ doping of CS. Furthermore, the conductivity and degradation of CS/PPy‐coated PLLA/PCL films were investigated, and their effect on neurite adhesion and elongation was evaluated via neural cell test.

## 2 MATERIALS AND METHODS

### 2.1 Preparation of electrospun PLLA/PCL

PLLA/PCL (75/25 w/w, Mv~230 000, see Data S1) block copolymer was dissolved in hexafluoroisopropanol (HFIP) as spinning solution with a concentration of 0.1 g/mL. The receiver was a cylinder with indium‐tin oxide (ITO) glass sheets (2 × 3 cm^2^), which could rotate with the roller at 500 rpm to collect the aligned fibres, as shown in Figure S1. The spinning time was 1.5 hours under 10 kV to obtain aligned PLLA/PCL fibre film on ITO sheet.

### 2.2 Preparation of conductive CS/PPy‐PLLA/PCL fibre films

PLLA/PCL fibres were coated with PPy and CS by electro‐deposition. First, CS (0.4 g) was dissolved in 1% of acetic acid solution (20 mL). Then, hydrochloric acid (0.2 mol/L, 30 mL) with pyrrole (97 μL)^37^ was added into the CS solution at 4°C and completely mixed as the pyrrole suspension. The ITO sheet with PLLA/PCL fibre film (2 × 3 cm^2^) as anode and the Au‐foil (2 × 3 cm^2^) as the cathode was immersed in the pyrrole suspension for electro‐deposition of PPy. After the deposition of 8 minutes under 2 mA of the current,^18^ the Cl^−^‐doped CS/PPy conductive shell was formed on PLLA/PCL fibre film. Subsequently, the composite fibre films were rinsed 3 times with deionized water before the fibre films were peeled off carefully from the ITO sheet. Finally, the obtained CS/PPy‐PLLA/PCL fibre films were dried at room temperature for 24 hours.

### 2.3 Characterization of CS/PPy‐PLLA/PCL fibre films

The morphology of the fibre films was observed with scanning electron microscopy (SEM, HITACHI S4800, Japan). The chemical composition of the samples was analysed with Fourier Transform Infrared Spectra (FTIR). In addition, there is a characteristic peak at 465 nm in absorption spectra to identify the interaction of Chitosan and Zincon (ZCN). Thus, the interaction was used to quantitatively analysed CS content by ultraviolet‐visible spectrophotometer.^38^, ^39^ Conductivity of the fibre films was measured with linear four‐point probe method (Qianfeng Electric Co., Shanghai, China). To investigate the film stability, CS/PPy‐PLLA/PCL and PPy‐PLLA/PCL fibre films (4 × 6 cm^2^) were cut into pieces of 0.2 × 1.5 cm^2^ and soaked in 0.9% NaCl solution (2 mL, pH = 6.89) for 0, 1, 2, 4, 8, 16 days (37°C); finally, the change of their weight (see Data S1) and the pH value of the immersion solution in different time were analysed. Two kinds of fibre films were also immersed in cell culture (RPMI 1640 medium supplemented with 10% foetal bovine serum and 5% horse serum at 37°C) for 0, 1, 2, 4, 8, 16 days; then, their tensile strength, conductivity and cyclic voltammetry (CV, Chenhua CHI660E, Shanghai, China) curve were measured. Tensile strength of the fibre films was measured with mechanical testing machine (SHIMADZU AG‐IC, Japan), and the fibre films was folded three times to form the sample with eight layers with the size of 2 × 0.4 cm^2^. In the measurement, the fibre films were stretched with the rate of 0.08 mm/s. CV curve is a type of potentiodynamic measurement^40^ to analyse the electrochemical property of fibre films.

### 2.4 Cell experiments of CS/PPy‐PLLA/PCL fibre films

#### 2.4.1 Cell compatibility

To investigate cyto‐compatibility of CS/PPy‐PLLA/PCL fibre film, a 3‐(4,5‐dimethylthiazol‐2‐yl)‐2,5‐ diphenyltetrazolium bromide (MTT) method was used to analyse the viability of L929 mouse fibroblast cells and PC12 cells. First, CS/PPy‐PLLA/PCL or PPy‐PLLA/PCL fibre films were placed in the 24‐well plate and disinfected by 75% alcohol for 0.5 hours and UV lights for 12 hours. Then, cells were cultured with CS/PPy‐PLLA/PCL or PPy‐PLLA/PCL in RPMI 1640 medium supplemented with 10% newborn calf serum for 1, 2 and 3 days at 37°C, 5% CO_2_ and humid atmosphere. Subsequently, MTT solution was added into each well and incubated at 37°C for 4 hours before the medium was replaced by DMSO. The cell viability (%) could be evaluated via the ratio of treated cells optical density (OD) to the control cells OD at 490 nm by micro plate reader (Bio‐Rad, 3550, USA). The cell viability (%) = optical density (OD) of the experimental cells/OD of the control cells. The test was repeated three times. The statistical significance of the treated L929 or PC12 cells with the control cells was determined by Tukey's post hoc of ANOVA.

#### 2.4.2 Cell differentiation and neurite growth

Undifferentiated PC12 cells purchased from Shanghai institute of biochemistry and biology were cultured in RPMI 1640 medium supplemented with 10% foetal bovine serum (Gibco) and 5% horse serum (Hyclone) at 37°C and 5% CO_2_. Prior to seeding, the cells were pre‐cultured with NGF of 50 ng/mL for 3 days to obtain their differentiation.^41^ At the same time, CS/PPy‐PLLA/PCL and PPy‐PLLA/PCL fibre films were placed in the 24‐well plate and disinfected with 75% alcohol for 0.5 hour and UV lights for 12 hours.

To observe the neurites from differentiated PC12 cells on two fibre films, pre‐cultured cells were cultured (1 × 10^4^ per well) with 50 ng/mL NGF and 50 ng/mL NT‐3 (Biovision) on these fibre films for 1, 3 and 5 days. In this process, NGF could maintain nerve survival, promote axon differentiation and improve nerve regeneration.^42^, ^43^ NT‐3 could maintain neuron growth and promote synapse formation.^44^ During the culture period, CS/PPy‐PLLA/PCL groups were exerted electrical stimulation (ES) with two electrodes (see Figure S2) at 100 mV for 2 hours every day.^45^ Then, phalloidin and 4′, 6‐diamidino‐2‐phenylindole (DAPI)‐stained cells on fibre films were observed under the inverted microscope. There were three parameters to be analysed as follows: cell differentiation rate, the neurite length and neurite alignment. (a) The cell with at least one neurite longer than the cell body was regarded as a differentiated cell.^46^, ^47^ The related protein, microtubule‐associated protein‐2 (MAP‐2), was measured by enzyme linked immunosorbent assay (ELISA) kit of MAP‐2. (b) The neurite length was measured from the tip of the neurite to the cell body by Image Pro Plus software. (c) The cell alignment was based on the angle between fibre axis and cell neurite. In this study, when electrical stimulation was exerted on cells with aligned fibre film, the electric field direction is parallel to the fibre axis. When the angle between fibre axis and cell neurite is less than 10°, the cell was regarded to be aligned along the fibre axis. These cells which aligned along the fibres were called “normalized cells”.

#### 2.4.3 Detection of proteins

Rat focal adhesion kinase (FAK), a kind of cytoplasmic protein tyrosine kinases, played an important role in the adhesion complexes and dynamic regulation of overall organization.^48^ FAK ELISA kit (ELISA LAB, Wuhan, China) was purchased to quantitatively determine FAK concentrations of PC12 cells cultured with various fibre films and different time of electrical stimulation of 100 mV (1, 3 and 5 days, 2 hours every day). Western blotting was a kind of immunochemical techniques, which was used to detect a specific protein immobilized on a matrix through electrophoresis.^49^ Sodium dodecyl sulphate polyacrylamide gel electrophoresis (SDS‐PAGE) was used to analyse the amount of neurofilament light polypeptide (NF‐L, one of subunits of neurofilament) and tyrosine kinase receptor A (Trk A, one of Trk receptors) with ChemiDoc XRS System with Image Lab Software (BIO‐RAD, USA). The neurofilament could form the neuronal cytoskeleton with microtubules and microfilaments, so NF‐L was involved in providing structural support for axons.^50^, ^51^ First, Trk A could bind NGF or NT‐3, and then, Trk receptors were able to regulate synaptic growth and cells differentiation through the activation pathway.^52^ Thus, the two proteins could be used to analyse the influence of electrical stimulation.

## 3 RESULTS

### 3.1 Composition, structure and property of CS/PPy‐PLLA/PCL fibre films

**Figure 1:**
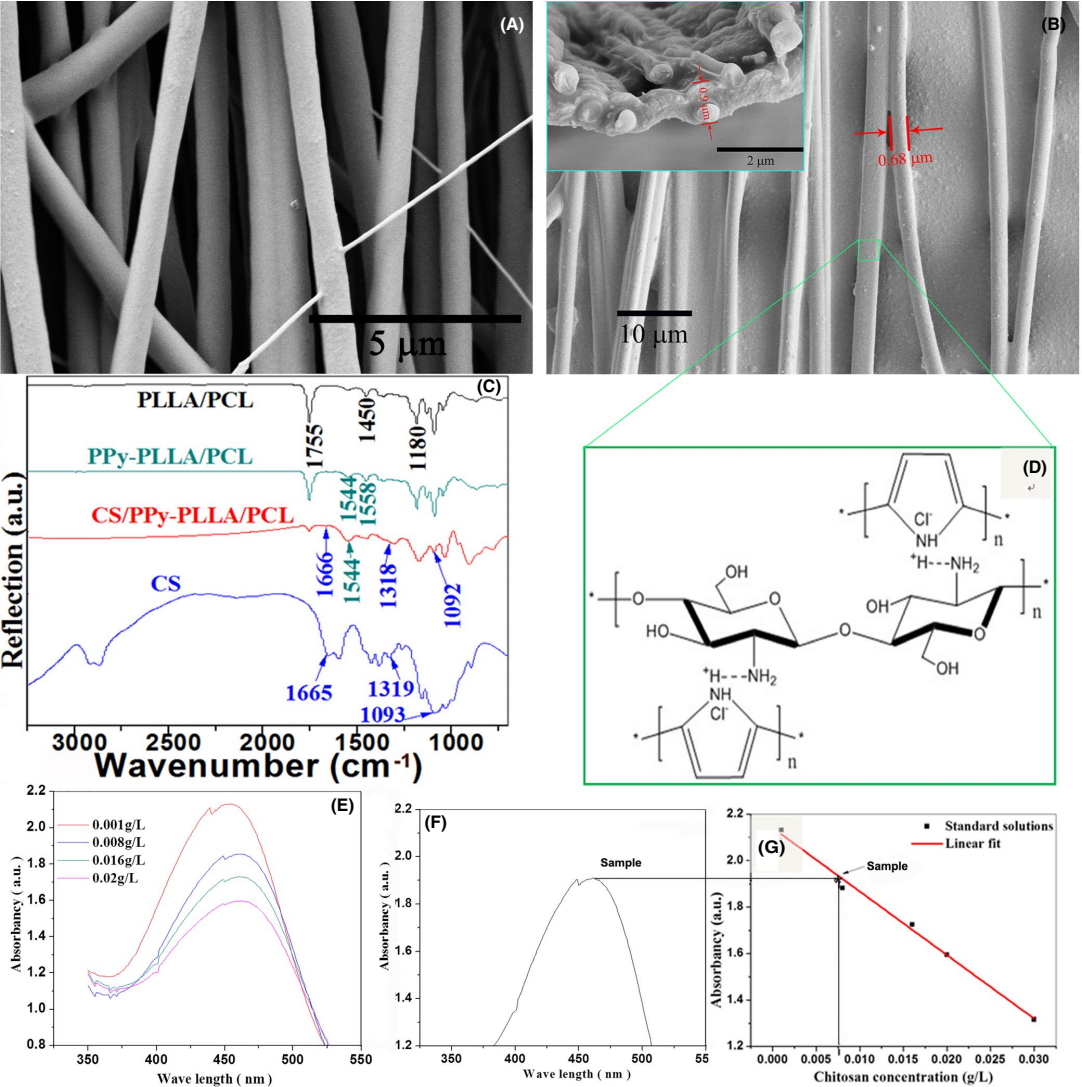
SEM images for the PLLA/PCL (A), CS/PPy‐PLLA/PCL fibre films and cross‐sectional view of CS/PPy‐PLLA/PCL fibre film (B). FTIR results for the CS particles, PLLA/PCL, PPy‐PLLA/PCL and CS/PPy‐PLLA/PCL fibre films (C). Schematic of conjugation of CS and Cl^−^‐doped PPy (D). Ultraviolet‐visible curves of solutions (E), sample (F) and the corresponding standard curve (G)

As shown in the image of Figure 1A, the electrospun PLLA/PCL fibres were aligned and almost paralleled to each other. Their average diameter was 0.57 μm. After CS/PPy was electro‐deposited on naked PLLA/PCL, the fibres in the deposition film remained aligned structure, as shown in Figure 1B. The average diameter of CS/PPy‐coated fibres reached to 0.68 μm. The cross‐sectional image of CS/PPy‐PLLA/PCL fibre film in Figure 1B shows that, the average thickness of the fibre film was about 0.9 μm. FTIR spectra of PLLA/PCL, PPy‐PLLA/PCL and CS/PPy‐PLLA/PCL fibre films in Figure 1C show that, the peaks at 1755, 1180 and 1450 cm^−1^ were C=O, C–O and C–H vibration of PLLA and PCL, respectively; the C–N and C=C stretching vibration peaks of pyrrole ring in PPy‐PLLA‐PCL pattern were located at 1458 and 1544 cm^−1^, respectively. The C=O and N–H stretching vibration peaks of the amide I and III in CS pattern were located at 1665 and 1319 cm^−1^, respectively, and C–O stretching vibration peak of the ether was located at 1093 cm^−1^. In the pattern of CS/PPy‐PLLA/PCL fibre film, there were weaker peaks at around 1666, 1318 and 1092, demonstrating that a few of CS chains were incorporated with PPy. Compared with the pattern of CS, these peaks slightly moved because of the decrease of the crystallinity of chitosan.^53^ Thus, the hydrogen bonds and van der Waals’ forces played a main role in the binding of PPy with CS (Figure 1D). These results demonstrated that CS was doped into the PPy shell. The results of Zincon analysis (Figure 1E‐G) further confirm the existence of CS in the conductive shell. Figure 1E,F showed the absorbance curves of the different concentration solutions of CS and the immersion solution of CS‐doped PPy shell in PPy/CS‐PCL/PLLA fibre films by ultraviolet‐visible spectrophotometer with the excitation wavelength of 465 nm. Figure 1G was the standard line established between the different concentrations of CS solutions and their absorbance. The fitted linear regression equation for the standard solutions was *y *= −27.348x + 2.1401, *R* ^2^ = 0.99147, for the quantitative analysis of CS in the PPy/CS‐PCL/PLLA fibre films. Therefore, the content of the CS on the as‐prepared PPy/CS‐PCL/PLLA fibre films is 7.5 mg/cm^2^.

**Figure 2:**
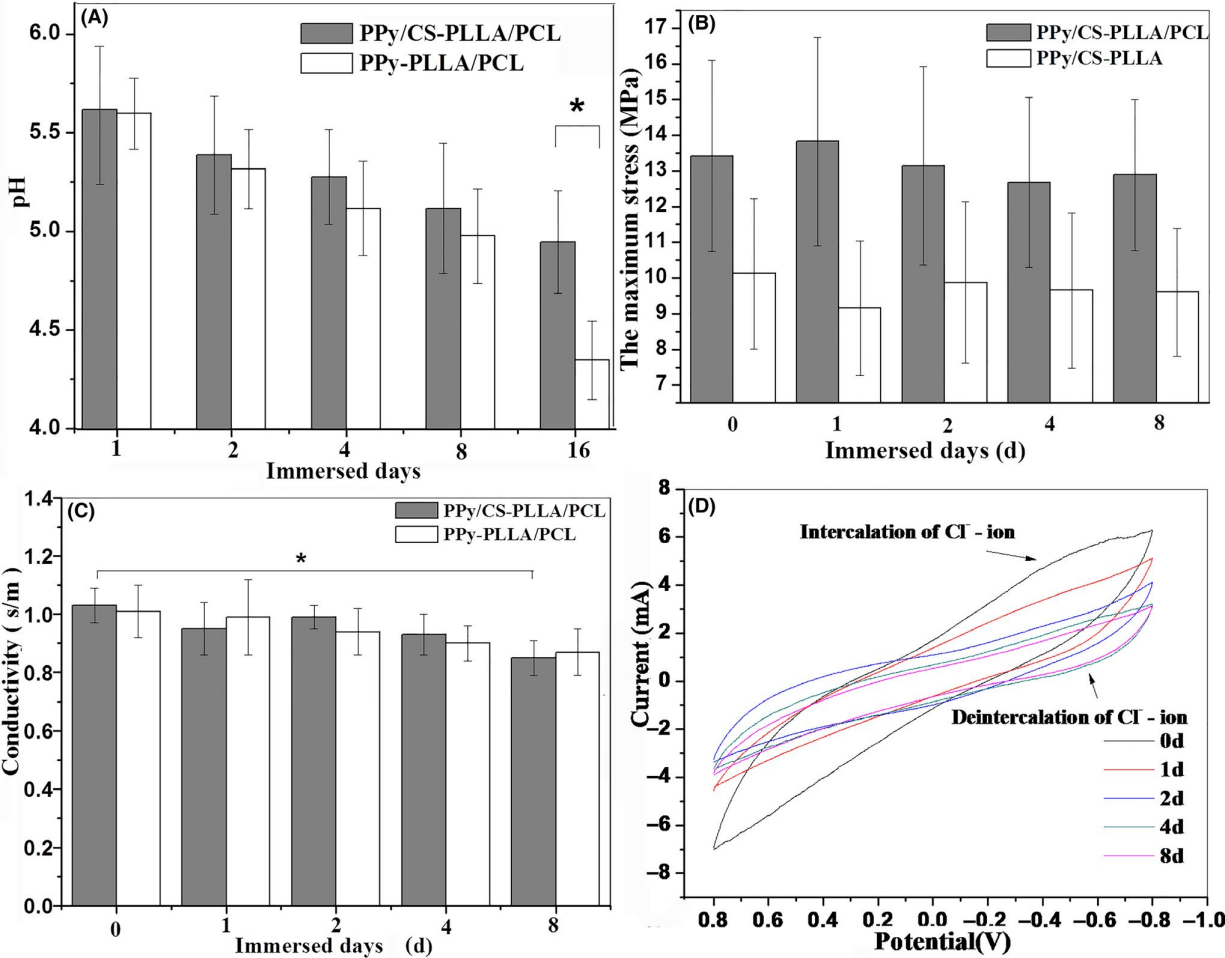
pH values of immersion solution (A), tensile strength (B) and conductivity (C) of CS/PPy‐PLLA/PCL and CS/PPy‐PLLA fibre films; CV curve of CS/PPy‐PLLA/PCL fibre films (D). The asterisk * indicates significant differences (*P* < 0.05) between two corresponding groups

As shown in Figure 2A, the pH values of two immersion solutions of CS/PPy‐PLLA/PCL and PPy‐PLLA/PCL fibre films decreased slowly in the first 8 days. However, the pH values from CS/PPy‐PLLA/PCL group were higher the corresponding values from PPy‐PLLA/PCL group. Furthermore, the pH value from CS/PPy‐PLLA/PCL group decreased to 5.1 at 16 days of immersion, significantly higher than ~4.3 of PPy‐PLLA/PCL group at 16 days (*P* < 0.05). These results suggest that CS/PPy‐PLLA/PCL fibre films were promising to reduce local aseptic inflammation risk caused by PLLA degradation, because the dedoped CS balanced pH value of solution. Hydrogen ions could induce the cleavage of ester bonds, accelerating the hydrolysis of PLLA and its copolymer, thus the dedoped CS could decrease the degradation rate of fibre films.^54^ The weight changes of fibre films immersed in 0.7% NaCl solution for 16 days are shown in Figure S4, indicating that the residue weights of PPy‐PLLA/PCL fibre films in 2nd‐16th day were less than those of CS/PPy‐PLLA/PCL fibre films. As shown in Figure 2B, there was no significant difference between the maximum stresses of two groups at the 16th day, meaning that the culture medium did not significantly affect tensile property for CS/PPy‐PLLA/PCL and CS/PPy‐PLLA fibrefilms. The maximum stress for CS/PPy‐PLLA/PCL fibre films levelled off at around 13.4 MPa, which was close to biomechanics of elbow joint ulnar nerve of rabbits,^55^ larger than around 9.15 MPa of CS/PPy‐PLLA fibre films. This result indicated that the addition of PCL increased the strength of copolymer fibres (see Figure S3). The results of Figure 2C show that the electrical conductivities of CS/PPy‐PLLA/PCL and PPy‐PLLA/PCL fibre films decreased slightly with the immersion time. The conductivity of the CS/PPy‐PLLA/PCL fibre films was 1.03 s/m before the immersion and could almost remain during the immersion of 4 days. After immersion for 8 days, Their conductivity significantly decreased to 0.85 s/m (*P* < 0.05), then continuously decreased to 0.75 s/m (*P* < 0.01) at the 16 days, suggesting that CS could dissolve into the solution, increasing conductive shells defects. CV curves in Figure 2D show that the electrochemical property of CS/PPy‐PLLA/PCL fibre film could maintain well stability for 16 days, because the charge‐discharge process of PPy coating corresponded with the adsorption and disadsorption of doped Cl^−^ ions, respectively.^56^ The weak peaks at −0.4 V and −0.5 V represented the intercalation and de‐intercalation behaviour of Cl^−^, respectively. In addition, the electric capacity of the prepared film was slowly decreased with the immersion time.

### 3.2 Cell evaluation on CS/PPy‐PLLA/PCL fibre films

#### 3.2.1 Cell compatibility

MTT results of L929 cells cultured on culture plates (control groups), CS/PPy‐PLLA/PCL and PPy‐PLLA/PCL fibre films are shown in Figure S5a. It is observed that the cyto‐viability in CS/PPy‐PLLA/PCL groups was significantly higher than that in control groups (*P* < 0.05), while PPy‐PLLA/PCL fibre films limited the cell viability (*P* < 0.01 or *P* < 0.05). The MTT results of PC12 cells (Figure S5b) show that the cyto‐viability in PPy‐PLLA/PCL groups was significantly lower than that of control groups at 3 days (*P* < 0.01), while the cyto‐viability in CS/PPy‐PLLA/PCL groups was significantly higher than that of control groups (*P* < 0.05). These results suggest that CS doping in the composite fibres could promote the viability of cells. Furthermore, there was significant difference between the two treated groups of films with/without CS (*P* < 0.01), meaning that CS could enhance the cyto‐compatibility of the prepared fibre films.

#### 3.2.2 Cell differentiation and neurite growth

**Figure 3:**
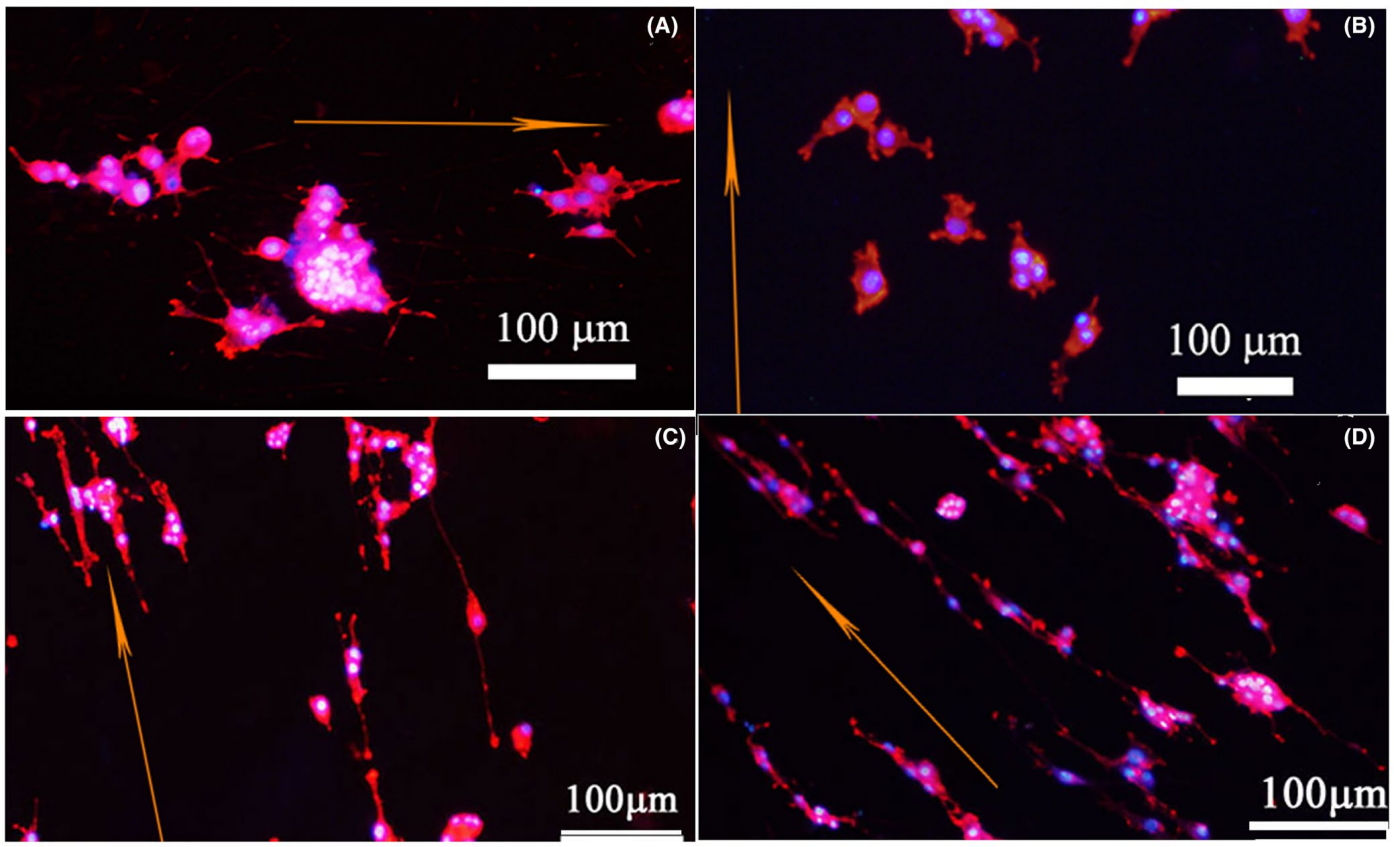
Immunofluorescence (phalloidin/DAPI) micrograph of PC12 cells cultured on different fibre films: (A, B) PPy‐PLLA/PCL, and (C, D) CS/PPy‐PLLA/PCL fibre films for 5 d, with electrical stimulation (ES) of 100 mV for 2 h every day (B, D) or without ES (A, C). The orange arrows represent direction of fibres axis

PC12 cells were cultured on CS/PPy‐PLLA/PCL and PPy‐PLLA/PCL fibre films with or without ES for 1, 3, 5 days, and the images are shown in Figure S6. Figure 3 is the images of PC12 cells cultured for 5 days on two kinds of fibre films with or without ES. The differentiated PC12 cells and the extended neurites could be observed clearly. The orange arrows represent direction of fibres axis. The neurites in each group were almost aligned to grow, and neurite length from the differentiated cells was increased with the cultivation time. Compared with ES groups (shown in Figure 3B,D), no ES groups (Figure 3A,C) showed less and shorter neurites. In addition, the cells cultured on CS/PPy‐PLLA/PCL fibre films outgrew more and longer neurites (Figure 3C,D), compared with those on PPy‐PLLA/PCL fibre films (Figure 3A,B). Especially, the image of a single cell cultured on CS/PPy‐PLLA/PCL fibre films in Figure S7 shows that the axon length was more than 100 μm and parallel with the direction of fibres axis.

**Figure 4:**
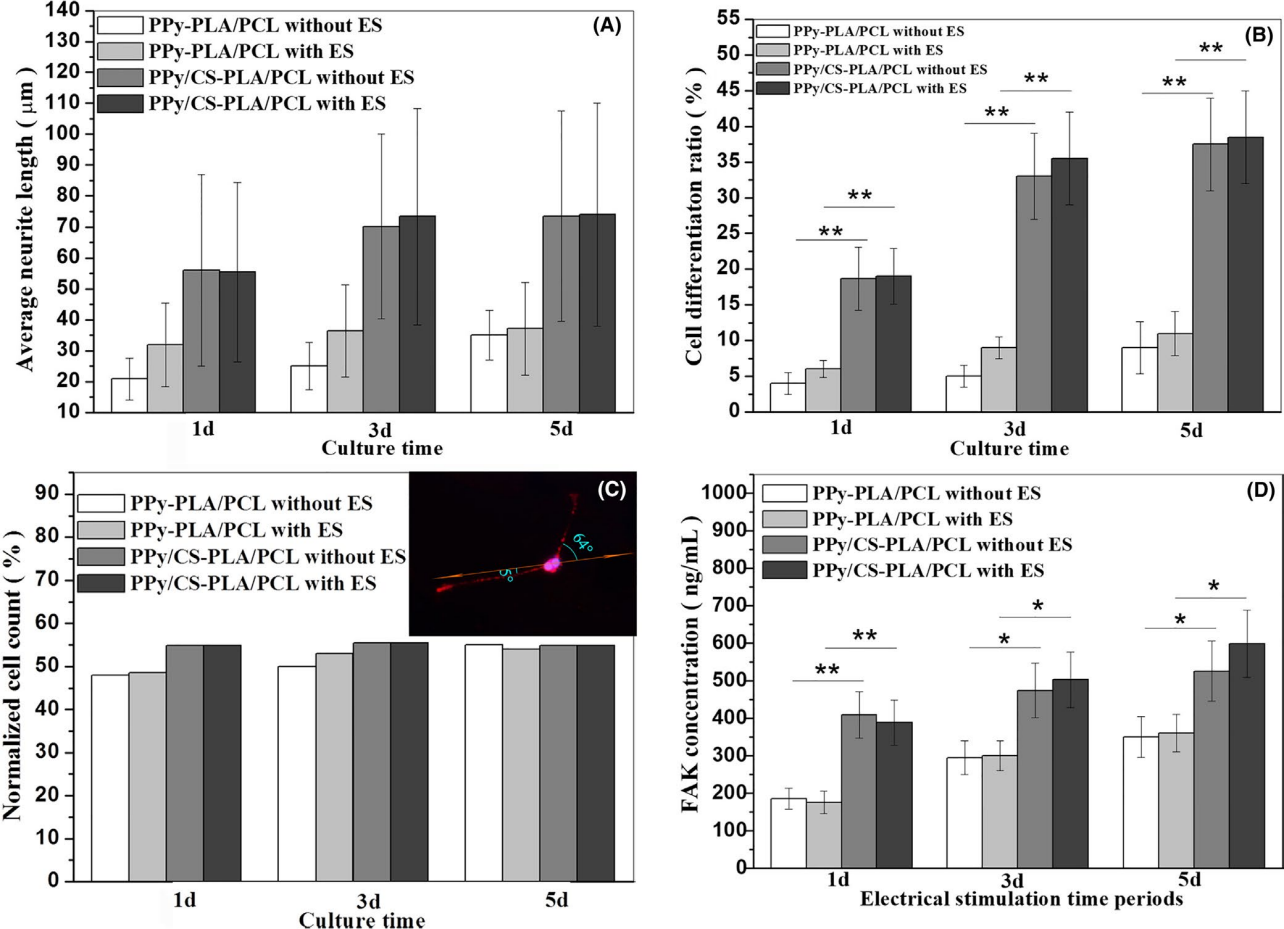
The histograms of neurites length (A), cell differentiation rate (B), normalized cell count (C) and FAK concentrations (D) of PC12 cells in different culture conditions for 1, 3 and 5 d. The asterisk *(*P* < 0.05) and **(*P* < 0.01) indicate positive significant differences between two groups. The immunofluorescence micrograph of PC12 cells in Figure 4C shows the angle between fibre axis and cell neurite

It is found in Figure 4A that, the neurite length of CS/PPy‐PLLA/PCL group was larger than PPy‐PLLA/PCL group (*P* < 0.01), and reached to about 75 μm at the 5th day. The differentiation rates (Figure 4B) of CS/PPy‐PLLA/PCL groups were significantly higher than group without CS (*P* < 0.01). The sketch map of angle between fibre axis and cell neurite are shown in inset of Figure 4C. At the first 3 days, the results of neurite alignment along the PPy‐PLLA/PCL fibre axis were slightly lower than CS/PPy‐PLLA/PCL groups. Then they tended to the same at the 5th day (about 55%), because the neurite length of PPy‐PLLA/PCL groups at the first 3 days (about 20‐30 μm, Figure 4A) were too short to extend along the fibres, leading to the unnoticeable orientation. In Figure 4D, the higher FAK concentration from CS/PPy‐PLLA/PCL group, compared with PPy‐PLLA/PCL group, demonstrated that CS in fibre films could support FAK protein production, thus improving the adhesion of PC12 cells on fibre films. To analyse the effect of electrical stimulation from fibre films on the cells, PC12 cells of experimental groups were placed into cell plates with fibre films and electrically stimulated for 1, 3 and 5 days, and 2 hours per day, while no electrical stimulation was used as control groups. As shown in Figure 4A, B, the results of neurite length and cell differentiation rate in the electrical stimulation group were slightly higher than those without ES, except for the neurite lengths results in the 1st day. These results suggested that, the electrical stimulation of 100 mV could slightly promote PC12 cells to protrude longer neurites. However, ES did not prominently influence the neurite alignment (Figure 4C). The results in Figure 4D show that the FAK concentration in ES group increased faster in the first 3 days (from ~387 pg/mL at 1 day to ~502 pg/mL at 3 days), then reaches to ~598 pg/mL at 5 days, higher than those of groups without ES, indicating that ES could improve adhesion of PC12.

**Figure 5:**
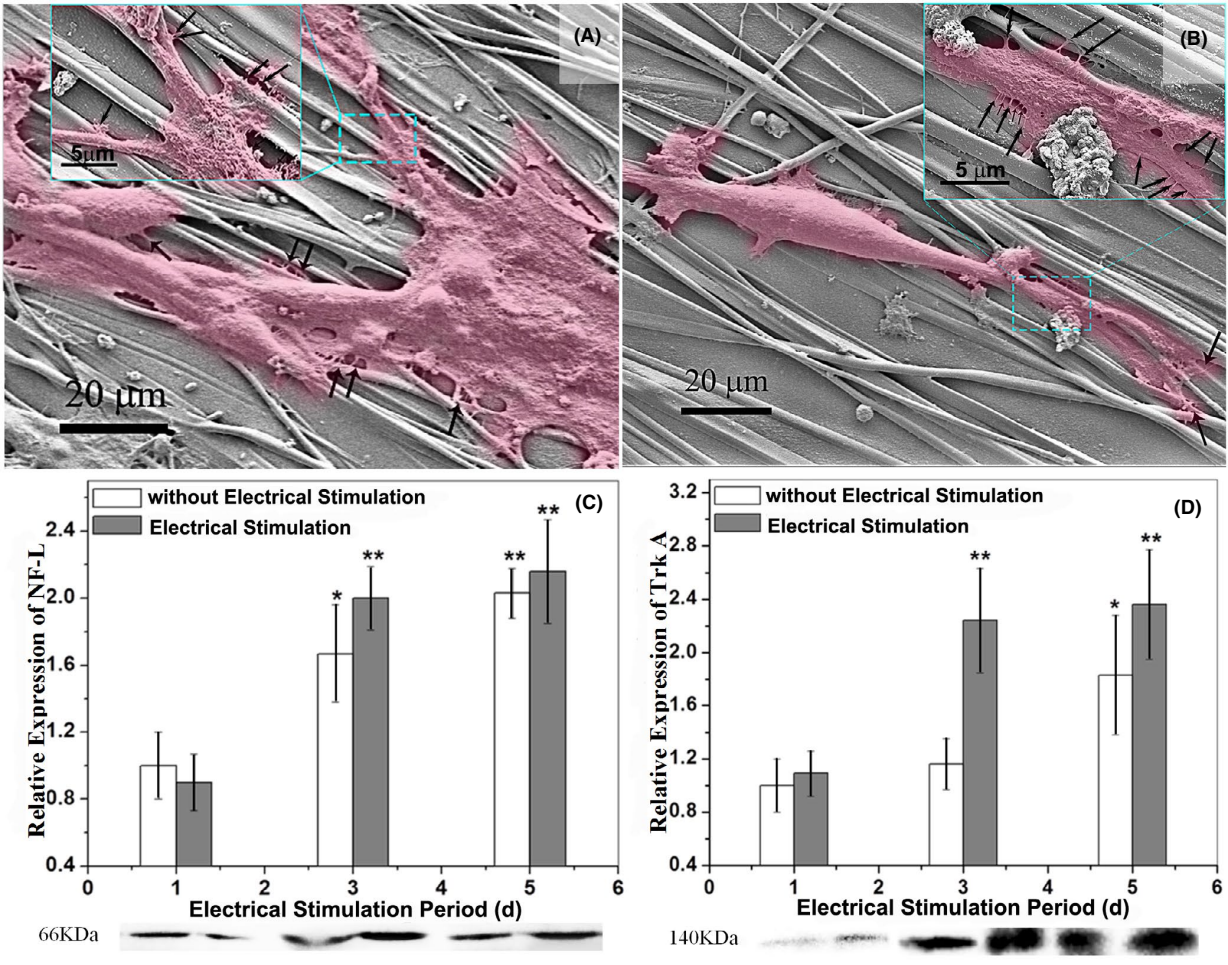
SEM images of PC12 cells cultured on CS/PPy‐PLLA/PCL fibre films for 3 days with (A) and without ES (B) are shown above. The PC12 cells are marked in pink to differentiate fibres from neurons. Histograms of relative expression of NF‐L (C) and Trk A (D) show the growing status of PC12 cells in different groups. The Western blotting results of NF‐L and Trk A are shown below the corresponding histograms. The asterisk *(*P* < 0.05) and **(*P* < 0.01) indicate positive significant differences compared with the group cultured 1 d without ES

Moreover, it is seen in SEM images of Figure 5A,B that, the fibrefilms maintain their aligned structures and smooth surfaces and could guide neurite growth along the fibre axis (see the magnified images of Figure 5A,B). Neurites attached to fibre surface or even grew into strip fibrous septa. As shown in Figure 5A, aligned fibre was the most important factor in guiding PC12 cells, compared with cultured time and ES time. Compared to no ES groups, the growth cones in ES groups were more elongated, moreover, there were more filopodia around growth cone in ES groups (marked by black arrows).

The amounts of NF‐L and Trk A (Figure 5C,D) increased rapidly in the first 3 days, showing significant differences, compared with those at the 1st day (*P* < 0.01), and then, there was a little increase at the 5rd day. These results further indicate that ES from fibre films could significantly improve differentiation of PC12 cells and axon outgrowth in the first 3 days.

## 4 DISCUSSION

**Figure 6:**
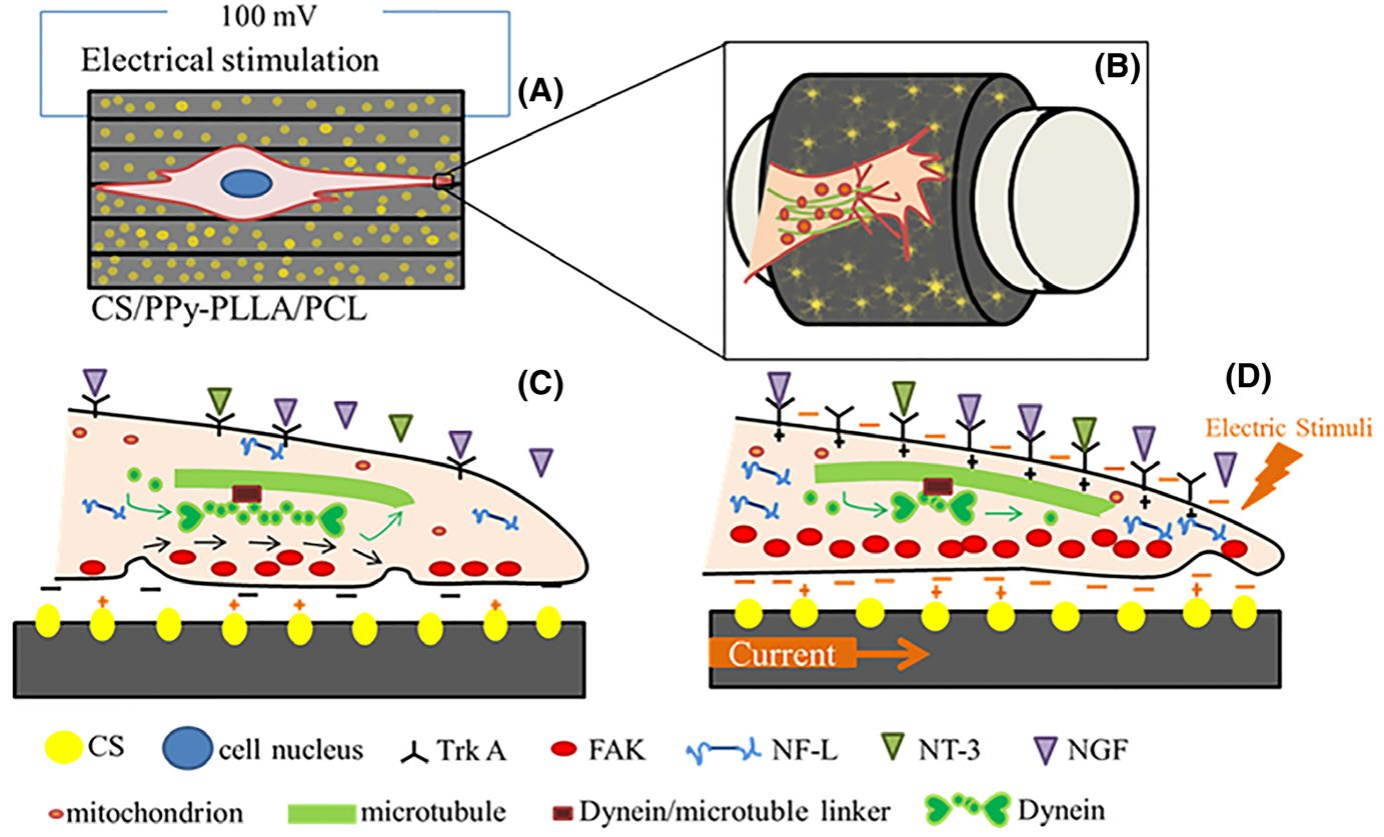
Schematic of PC12 cells cultured on CS/PPy‐PLLA/PCL fibres film under electrical stimulation (A): composited aligned fibres could guide axon growth (B); Cross‐section of a filopodium extension of growing axon before (C) and after (D) electrical stimulation through the expression of FAK, NF‐L and Trk A

Based on the above results, a synergistic mechanism about effect of CS, ES and aligned fibres on PC12 cells adhesion, axon outgrowth is proposed. The schematic of PC12 cells cultured on CS/PPy‐PLLA/PCL fibre film under ES is shown in Figure 6A. The filopodia could perceive extracellular guidance cues^57^, ^58^ and extend along fibres (Figure 6B). Because the exposed CS chains have positive charge, they can be combined with cell membrane with negative charge to promote cells adherence (Figure 6C). When NGF and NT‐3 stimulate PC12, they can phosphorylate Trk A and enhance catalytic activity of Trk A, leading to the activation of signalling cascades. Especially NGF/Trk A coupling can induce preferentially the activation pathway, thus resulting in cells differentiation and axon outgrowth.^52^, ^59^, ^60^ To continually extend, the microtubules in filopodia are coupled with the dynein via dynein/microtubule linkers^61^, ^62^, ^63^ and repeated cycles of self‐assembly and disassembly^57^ (marked by green arrows in Figure 6c). In this process, growth cone collapsed at the neck to form new cytoplasmic domain of the axon (marked by black arrows).^57^ Neurofilament protein (NF) was closely related to the acceleration of axoplasmic transport.^56^ The amounts of three kinds of proteins under ES were slightly increased, compared with those without ES (Figure 6D and 5C,D). Although cell membranes obtain negative charge under ES, they can be combined well with positive charges of CS, and the adhesion receptors on cell membranes can easily anchor on the fibre surface.^64^, ^65^ Then more FAK transduce the anchor signal to cell,^65^ increasing cells adherence^66^ (Figure 6D). At the same time, receptors of TrkA in cell membrane induced by electrical stimulation^67^ could receive more NGF and NT‐3. With effect of NGF and NT‐3, the microtubules in filopodia and cytoplasmic domain move to the end of the axon, leading to the increase of NF‐L expression. In addition, ES provides energy to the dynein assembly and disassembly,^57^, ^68^ resulting in axon outgrowth. Thus, when PC12 cells are exerted with ES, they would produce galvanotaxis.^69^ With the increase of FAK, Trk A and NF‐L, PC12 cells present better differentiation and axon outgrowth.

## 5 CONCLUSIONS

CS/PPy‐PLLA/PCL fibre films with good cyto‐compatibility were prepared through the electro‐deposition of CS‐doped PPy on aligned PLLA/PCL fibre films. The content of CS on the conductive shell was 7.5 mg/cm^2^. The tensile strength of this fibre film was around 13 MPa, which was close to that of elbow joint ulnar nerve of rabbits.^48^ After the immersion in saline for 16 days, the immersion solution of CS/PPy‐PLLA/PCL fibre films could still maintain stable pH of about 5.1 (higher than 4.7 of the sample without CS), indicating that CS doping could reduce local acid property from the degradation product. Due to its surface conductivity of about 1.03 s/m, ES was available to promote the growth and differentiation of PC12 cells, and to support the directional growth of neurites. A mechanism on the synergistic effect of CS, aligned fibre films and ES to promote cell adhesion and axon growth was proposed. Therefore, the CS/PPy‐coated PLLA/PCL fibre films would be applicable to the nerve tissue engineering in future.

## References

[1] Pham QP , Sharma U , Mikos AG . Electrospinning of polymeric nanofibers for tissue engineering applications: a review. Tissue Eng. 2006;12:1197‐1211. 16771634 10.1089/ten.2006.12.1197

[2] Mooney DJ , Park S , Kaufmann PM . Biodegradable sponges for hepatocyte transplantation. J Biomed Mater Res. 1995;29:959‐965. 7593039 10.1002/jbm.820290807

[3] Schugens C , Grandfils C , Jerome R , et al. Preparation of a macroporous biodegradable polylactide implant for neuronal transplantation. J Biomed Mater Res. 1995;29:1349‐1362. 8582903 10.1002/jbm.820291106

[4] Kim BS , Mooney DJ . Development of biocompatible synthetic extracellular matrices for tissue engineering. Trends Biotechnol. 1998;16:224‐230. 9621462 10.1016/s0167-7799(98)01191-3

[5] Qiaozhen Y , Shuiling X , Kuihua Z . Multi‐porous electroactive poly(L‐lactic acid)/polypyrrole composite micro/nano fibrous scaffolds promote neurite outgrowth in PC12 cells. Neural Regen Res. 2013;8:31‐38. 25206369 10.3969/j.issn.1673-5374.2013.01.004 PMC4107503

[6] Heath CA , Rutkowski GE . The development of bioartificial nerve grafts for peripheral‐nerve regeneration. Trends Biotechnol. 1998;16:163‐168. 9586239 10.1016/s0167-7799(97)01165-7

[7] Zupancic S , Kocbek P , Baumgartner S . Contribution of nanotechnology to improved treatment of periodontal disease. Curr Pharm Design. 2015;21:3257‐3271. 10.2174/1381612821666150531171829 26027560

[8] Evans GR , Brandt K , Niederbichler AD . Clinical long‐term in vivo evaluation of poly(L‐lactic acid) porous conduits for peripheral nerve regeneration. J Biomat Sci‐Polym E. 2000;11:869‐878. 10.1163/156856200744066 11211097

[9] Yang F , Murugan R , Wang S . Electrospinning of nano/micro scale poly(L‐lactic acid) aligned fibers and their potential in neural tissue engineering. Biomaterials. 2005;26:2603‐2610. 15585263 10.1016/j.biomaterials.2004.06.051

[10] Zhou JF , Wang YG , Liang C , et al. Preparation of polypyrrole‐embedded electrospun poly(lactic acid) nanofibrous scaffolds for nerve tissue engineering. Neural Regen Res. 2016;11:1644‐1652. 27904497 10.4103/1673-5374.193245 PMC5116845

[11] Xie J , Macewan MR , Willerth SM , et al. Conductive core‐sheath nanofibers and their potential application in neural tissue engineering. Adv Funct Mater. 2009;19:2312‐2318. 19830261 10.1002/adfm.200801904 PMC2760838

[12] Ateh DD , Navsaria HA , Vadgama P . Polypyrrole‐based conducting polymers and interactions with biological tissues. J R Soc Interf. 2006;3:741‐752. 10.1098/rsif.2006.0141 PMC1885362 17015302

[13] Lee JY . Electrically conducting polymer‐based nanofibrous scaffolds for tissue engineering applications. Polym Rev. 2013;53:443‐459.

[14] Williams RL , Doherty PJ . A preliminary assessment of poly(pyrrole) in nerve guide studies. J Mater Sci. 1994;5:429‐433.

[15] Rivers TJ , Hudson TW , Schmidt CE . Synthesis of a novel, biodegradable electrically conducting polymer for biomedical applications. Adv Funct Mater. 2002;12:33‐37.

[16] George PM , Saigal R , Lawlor MW . Three‐dimensional conductive constructs for nerve regeneration. J Biomed Mater Res A. 2009;91:519‐527. 18985787 10.1002/jbm.a.32226

[17] Rutella S , Bonanno G , Procoli A , et al. Cells with characteristics of cancer stem/progenitor cells express the CD133 antigen in human endometrial tumors. Clin Cancer Res. 2009;15:4299‐4311. 19509143 10.1158/1078-0432.CCR-08-1883

[18] Zhou XX , Yang AN , Huang ZB , et al. Enhancement of neurite adhesion, alignment and elongation on conductive polypyrrole‐poly(lactide acid) fibers with cell‐derived extracellular matrix. Colloid Surface B. 2017;149:217‐225. 10.1016/j.colsurfb.2016.10.014 27768911

[19] Shafei S , Foroughi J , Stevens L , et al. Electroactive nanostructured scaffold produced by controlled deposition of PPy on electrospun PCL fibres. Res Chem Intermed. 2017;43:1235‐1251.

[20] Tia L , Prabhakaran MP , Hu J , et al. Synergistic effect of topography, surface chemistry and conductivity of the electrospun nanofibrous scaffold on cellular response of PC12 cells. Colloid Surface B. 2016;145:420‐429. 10.1016/j.colsurfb.2016.05.032 27232305

[21] Irani S , Zandi M , Salamian N , et al. The study of P19 stem cell behavior on aligned oriented electrospun poly(lactic‐co‐glycolic acid) nano‐fibers for neural tissue engineering. Polym Adv Technol. 2014;25:562‐567.

[22] Xia XL , Liu WT , Wang LN , et al. A Review of Biomedical Poly(lactic acid) Modification. Research Progress Polym Bull 2013;49:29‐38.

[23] Finotti PFM , Costa LC , Chinelatto MA . Effect of the chemical structure of compatibilizers on the thermal, mechanical and morphological properties of immiscible pla/pcl blends. Macromol Symp. 2016;368:24‐29.

[24] Mittal V , Akhtar T , Luckachan G . PLA, TPS and PCL binary and ternary blends: structural characterization and time‐dependent morphological changes. Colloid Polym Sci. 2015;293:573‐585.

[25] Ghasemi‐Mobarakeh L , Prabhakaran MP , Morshed M , et al. Bio‐functionalized PCL nanofibrous scaffolds for nerve tissue engineering. Mater Sci Eng, C. 2010;30:1129‐1136.

[26] Koh HS , Yong T , Chan CK , et al. Enhancement of neurite outgrowth using nano‐structured scaffolds coupled with laminin. Biomaterials. 2008;29:3574‐3582. 18533251 10.1016/j.biomaterials.2008.05.014

[27] Kim C , Choi E , Park J . Effect of PEG molecular weight on the tensile toughness of starch/PCL/PEG blends. J Appl Polym Sci. 2015;77:2049‐2056.

[28] Bai H , Hao X , Gao J . Tailoring impact toughness of Poly(l‐lactide)/Poly(ε‐caprolactone) (PLLA/PCL) blends by controlling crystallization of PLLA matrix. ACS Appl Mater Inter. 2012;4:897‐905. 10.1021/am201564f 22214560

[29] Cooper A , Bhattarai N , Zhang M . Fabrication and cellular compatibility of aligned chitosan–PCL fibers for nerve tissue regeneration. Carbohyd Polym. 2011;85:149‐156.

[30] Jian D , Tan E , Kim HJ , et al. Comparative evaluation of chitosan, cellulose acetate, and polyethersulfone nanofiber scaffolds for neural differentiation. Carbohyd Polym. 2014;99:483‐490. 10.1016/j.carbpol.2013.08.050 PMC3856206 24274534

[31] Bell JH , Haycock JW . Next generation nerve guides: materials, fabrication, growth factors, and cell delivery. Tissue Eng Part B Rev. 2012;18:116‐128. 22010760 10.1089/ten.TEB.2011.0498

[32] Patel M , Vandevord PJ , Matthew H , et al. Video‐gait analysis of functional recovery of nerve repaired with chitosan nerve guides. Tissue Eng Part A Rev. 2006;12:3189‐3199. 10.1089/ten.2006.12.3189 17518633

[33] Wang X , Hu W , Cao Y , et al. Dog sciatic nerve regeneration across a 30‐mm defect bridged by a chitosan/PGA artificial nerve graft. Brain. 2005;128:1897‐1910. 15872018 10.1093/brain/awh517

[34] Zhao Y , Wang Y , Gong J , et al. Chitosan degradation products facilitate peripheral nerve regeneration by improving macrophage‐constructed microenvironments. Biomaterials. 2017;134:64‐77. 28456077 10.1016/j.biomaterials.2017.02.026

[35] Lehr CM , Bouwstra JA , Schacht EH , et al. In vitro evaluation of mucoadhesive properties of chitosan and some other natural polymers. Int J Pharm. 1992;78:43‐48.

[36] Freier T , Koh HS , Kazazian K , et al. Controlling cell adhesion and degradation of chitosan films by N‐acetylation. Biomaterials. 2005;26:5872‐5878. 15949553 10.1016/j.biomaterials.2005.02.033

[37] Jin J , Huang ZB , Yin GF , et al. Fabrication of polypyrrole/proteins composite film and their electro‐controlled release for axons outgrowth. Electrochim Acta. 2015;185:172‐177.

[38] Gao GA , Jiao QA , Ding YA , et al. A Study on the mechanism of the interaction between chitosan and zincon. Chinese J Chem Phys. 2003;16:331‐336.

[39] Chi Y , Zhuang J , Li N , et al. Studies on the reaction between zincon bovine serum albumin. Chem Res Chinese. 1999;20:1697‐1702.

[40] Elgrishi N , Rountree KJ , Mccarthy BD . A practical beginner's guide to cyclic voltammetry. J Chem Educ. 2018;95:197‐206.

[41] Van Buskirk RG , Gabriels J , Wagner J . PC12 cells grown on cellulosic filters differentiate in response to NGF and exhibit a polarity not seen when they are grown on solid substrata. Vitro Cell Dev‐Pl. 1988;24:451‐456. 10.1007/BF02628497 3372449

[42] Heumann R , Lindholm D , Bandtlow C , et al. Differential regulation of mRNA encoding nerve growth factor and its receptor in rat sciatic nerve during development, degeneration, and regeneration: role of macrophages. P Natl Acad Sci USA. 2010;23:665‐669. 10.1073/pnas.84.23.8735 PMC299621 2825206

[43] Tuszynski MH , Gabriel K , Gage FH , et al. Nerve growth factor delivery by gene transfer induces differential outgrowth of sensory, motor, and noradrenergic neurites after adult spinal cord injury. Exp Neurol. 1996;137:157‐173. 8566208 10.1006/exnr.1996.0016

[44] Maisonpierre PC , Belluscio L , Squinto S , et al. Neurotrophin‐3: a neurotrophic factor related to NGF and BDNF. Science. 1990;247:1446‐1451. 2321006 10.1126/science.247.4949.1446

[45] Schmidt CE , Shastri VR , Vacanti JP , et al. Stimulation of neurite outgrowth using an electrically conducting polymer. P Natl Acad Sci USA. 1997;94:8948‐8953. 10.1073/pnas.94.17.8948 PMC22977 9256415

[46] Takatsuki H , Sakanishi A . Regulation of neurite outgrowth by extracellular Ca for neural cells PC12 and PC12D. Colloid Surface B. 2003;32:69‐76.

[47] Stiegler NV , Krug AK , Matt F . Assessment of chemical‐induced impairment of human neurite outgrowth by multiparametric live cell imaging in high‐density cultures. Toxicol Sci. 2011;121:73‐87. 21342877 10.1093/toxsci/kfr034

[48] Hsia DA , Mitra SK , Hauck CR , et al. Differential regulation of cell motility and invasion by FAK. J Cell Biol. 2003;160:753‐767. 12615911 10.1083/jcb.200212114 PMC2173366

[49] Millar T , Knighton R , Chuck JA . Using biotinylated proteins to demonstrate immunodetection of antigens via western blotting, dot blots, and immunohistochemistry. Methods Mol Biol. 2015;1314:151‐164. 26139263 10.1007/978-1-4939-2718-0_16

[50] Brureau A , Blanchard‐Bregeon V , Pech C , et al. NF‐L in cerebrospinal fluid and serum is a biomarker of neuronal damage in an inducible mouse model of neurodegeneration. Neurobiol Dis. 2017;104:73‐84. 28392472 10.1016/j.nbd.2017.04.007

[51] Löhrke S , Brandstätter JH , Boycott BB , et al. Expression of neurofilament proteins by horizontal cells in the rabbit retina varies with retinal location. J Neurocytol. 1995;24:283‐300. 7543937 10.1007/BF01186541

[52] Segal RA . Selectivity in neurotrophin signaling: theme and variations. Annu Rev Neurosci. 2003;26:299‐330. 12598680 10.1146/annurev.neuro.26.041002.131421

[53] Hughes J , Ramsden DK , Symes KC . The flocculation of bacteria using cationic synthetic flocculants and chitosan. Biotechnol Tech. 1990;4:55‐60.

[54] Tsuji H , Ikarashi K . In vitro hydrolysis of poly(‐lactide) crystalline residues as extended‐chain crystallites. Part III: effects of pH and Enzyme. Polym Degrad Stabil 2004;85:647‐656.

[55] Shen Z , Yang YG , Gao M . Experimental study on biomechanics of elbow joint ulnar nerve on fluorosis of bone. Chin J Ctrl Endem Dis. 2008;23:25‐26.

[56] Weidlich C , Mangold KM , Jüttner K . EQCM study of the ion exchange behaviour of polypyrrole with different counterions in different electrolytes. Electrochim Acta. 2005;50:1547‐1552.

[57] Suter DM , Miller KE . The emerging role of forces in axonal elongation. Prog Neurobiol. 2011;94:91‐101. 21527310 10.1016/j.pneurobio.2011.04.002 PMC3115633

[58] Suter DM , Forscher P . Substrate‐cytoskeletal coupling as amechanism for the regulation of growth cone motility and guidance. J Neurobiol. 2000;44:97‐113. 10934315

[59] Huang EJ , Reichardt LF . Trk receptors: roles in neuronal signal transduction. Annu Rev Biochem. 2003;72:609‐642. 12676795 10.1146/annurev.biochem.72.121801.161629

[60] Kamei N , Tanaka N , Oishi Y . BDNF, NT‐3, and NGF released from transplanted neural progenitor cells promote corticospinal axon growth in organotypic cocultures. Spine. 2007;32:1272‐1278. 17515814 10.1097/BRS.0b013e318059afab

[61] Charest JL , Eliason MT , García AJ , et al. Combined microscale mechanical topography and chemical patterns on polymer cell culture substrates. Biomaterials. 2006;27:2487‐2494. 16325902 10.1016/j.biomaterials.2005.11.022

[62] Vaudry D , Stork PJ , Lazarovici P , et al. Signaling pathways for PC12 cell differentiation: making the right connections. Science. 2002;296:1648‐1649. 12040181 10.1126/science.1071552

[63] Lamoureux P , Heidemann SR , Martzke NR , et al. Miller. Growth and elongation within and along the axon. Dev Neurobiol. 2010;70:135‐149. 19950193 10.1002/dneu.20764

[64] Yang AN , Huang ZB , Yin GF , et al. Fabrication of aligned, porous and conductive fibers and their effects on cell adhesion and guidance. Colloid Surface B. 2015;134:469‐474. 10.1016/j.colsurfb.2015.07.028 26258750

[65] Holmes WR , Park J , Levchenko A , et al. A mathematical model coupling polarity signaling to cell adhesion explains diverse cell migration patterns. PLoS Comput Biol. 2017;13:e1005524. 28472054 10.1371/journal.pcbi.1005524 PMC5436877

[66] Pérez E , Muñoz ML , Ortega A . Entamoeba histolytica: involvement of pp125FAK in collagen‐induced signal transduction. Exp Parasitol. 1996;82:164‐170. 8617343 10.1006/expr.1996.0021

[67] Chen PR , Chen MH , Lin FH , et al. Release characteristics and bioactivity of gelatin‐tricalcium phosphate membranes covalently immobilized with nerve growth factors. Biomaterials. 2005;26:6579‐6587. 16023717 10.1016/j.biomaterials.2005.03.037

[68] Geremia NM , Gordon T , Brushart TM , et al. Electrical stimulation promotes sensory neuron regeneration and growth‐associated gene expression. Exp Neurol. 2007;205:347‐359. 17428474 10.1016/j.expneurol.2007.01.040

[69] Fang KS , Ionides E , Oster G , et al. Epidermal growth factor receptor relocalization and kinase activity are necessary for directional migration of keratinocytes in DC electric fields. J Cell Sci. 1999;112:1967‐1978. 10341215 10.1242/jcs.112.12.1967

