# Supplementary material for: Fabrication of Chitosan/Polypyrrole‐coated poly(L‐lactic acid)/Polycaprolactone aligned fibre films for enhancement of neural cell compatibility and neurite growth
Source: Cell Prolif. 2019 Apr 11;52(3):e12588. doi: 10.1111/cpr.12588 (PMC6536449; doi:10.1111/cpr.12588)
Supplement: Supplementary file 1 [file CPR-52-e12588-s001.docx]

**Supplementary Material:**

**Fabrication of Chitosan/Polypyrrole-Coated Poly(L-lactic acid)/Polycaprolactone Aligned Fiber-films for the Enhancement of Neural Cell Cyto-Compatibility and Neurite Growth**

Yaxuan Xu, Zhongbing Huang∗, Guangfu Yin, Ximing Pu, Jiankai Zhang

College of Materials Science and Engineering, Sichuan University, Chengdu, China.

Address: No.24, South 1st Section, 1st Ring Road, Chengdu, 610065, China

Email: [zbhuang@scu.edu.cn](mailto:zbhuang@scu.edu.cn)

**Correspondence:**

Zhongbing Huang, College of Materials Science and Engineering, Sichuan University,

Address: No.24, South 1st Section, 1st Ring Road, Chengdu, 610065, China,

Email: zbhuang@scu.edu.cn; Fax: 86-28-85413003; Tel: 86-28-85413003

1. **Materials**

Pyrrole (Py, 99%) were obtained from Kelong Chemical Co. and used without further purifications. Poly(L-lactic acid) and poly(ε-caprolactone) copolymer (PLLA/PCL, 75/25 w/w, 2.5 dL/g) were purchased from Shandong institute of medical instruments. Exafluoroisopropanol (HFIP) was obtained from Aladdin Industrial Co., and chitosan (CS) were purchased from Shenzhen Zhongfayuan Biological Technology Co., LTD.

As shown in Figure S1, to obtain electrospun nanofibers, the PLLA/PCL (75/25 w/w) copolymer were dissolved in hexafluoroisopropanol (HFIP) as spinning solution with a concentration of 1 g·mL^‒1^. When high voltage of 10 kV was exerted, the Taylor cone was formed at spinneret of syringe. Then PLLA/PCL nanofibers were drawn constantly. The receiver was a cylinder with Indium-Tin Oxide (ITO) glass sheets (2 × 3 cm^2^), which could rotate at 800 rpm to collect the aligned fibbers. The spinning time was 1.5 h under 10 kV to obtain ITO sheets with aligned PLLA/PCL fibers.

**FIGURE S1.** Schematic of electrostatic spinning.


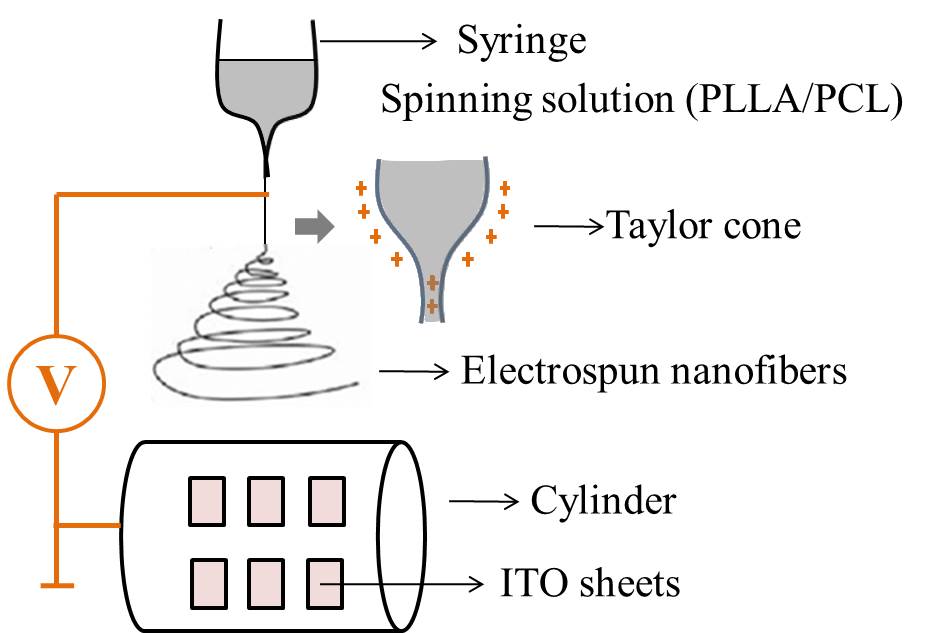


Figure S2 shows the schematic of electrical stimulation in cell culture. The composite fibers-film was immobilized between glass slide and glass well by polydimethylsiloxane (PDMS), and PC12 cells are cultured in the glass well (Φ = 10 mm, φ = 8 mm, and height = 10 mm). Then the electrical stimulation of 100 mV was exerted on the conductive fibers-film.

**FIGURE S2.** Schematic of cell cultured under electrical stimulation.


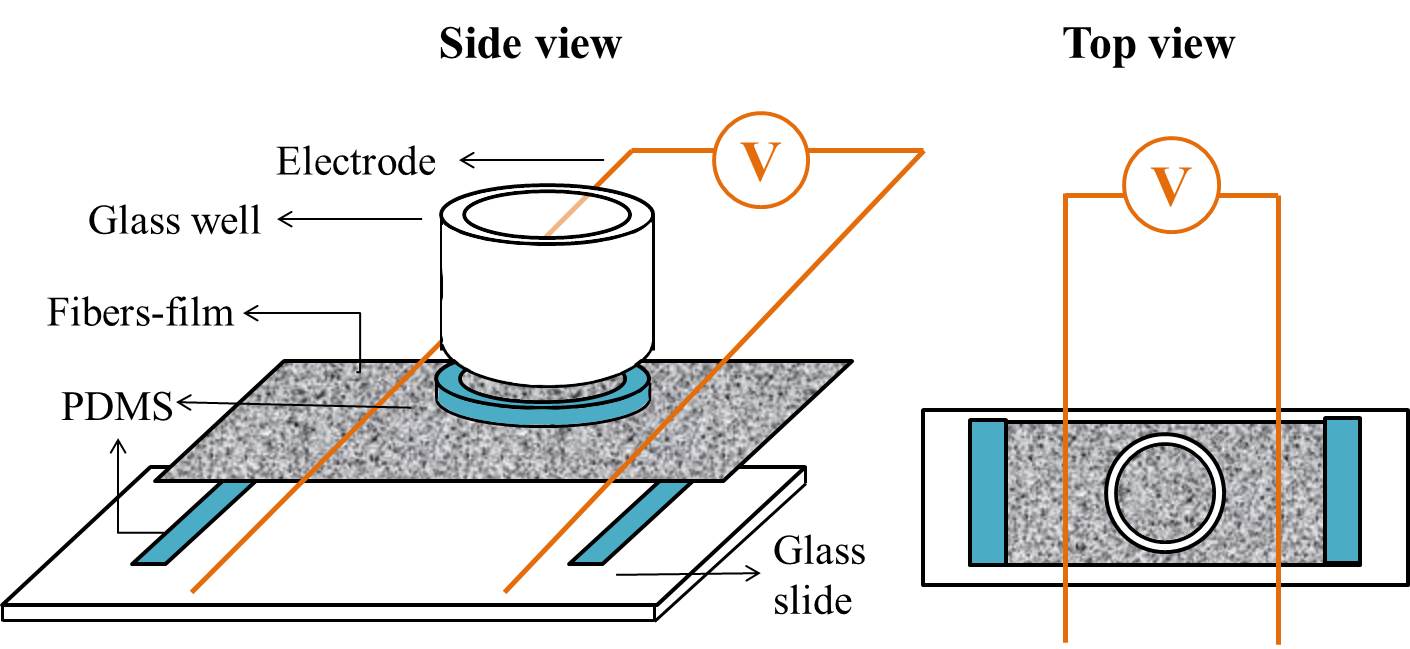


**2. X-Ray diffraction of fiber-films**

X-Ray diffraction (XRD, Fangyuan DX-1000, Dandong, China) were used to evaluate the crystal structure of the aligned fiber-films. Figure S3 shows XRD patterns of PPy/CS-PLLA, PPy-PLLA/PCL and PPy/CS- PLLA/PCL fiber-films. It is seen that there was no discernible diffraction peak in PPy/CS-PLLA fiber-films. However, the diffraction peaks of PPy/CS-PCL/PLLA fiber-films were at 16.67^o^ and 16.89^o^, meaning the slight crystal structure of PPy/CS-PCL/PLLA fiber-films ^1^. That is to say, the addition of PCL increased the crystallinity of copolymer fibers. The weaker crystal peaks still existed in the pattern of PPy-PCL/PLLA fiber-films (16.58^o^ and 16.95^o^), meaning that CS has no impact on crystal structure.

**FIGURE S3.** XRD results for the PPy/CS- PLLA/PCL, PPy-PLLA/PCL and PPy/CS-PLLA fiber-films.


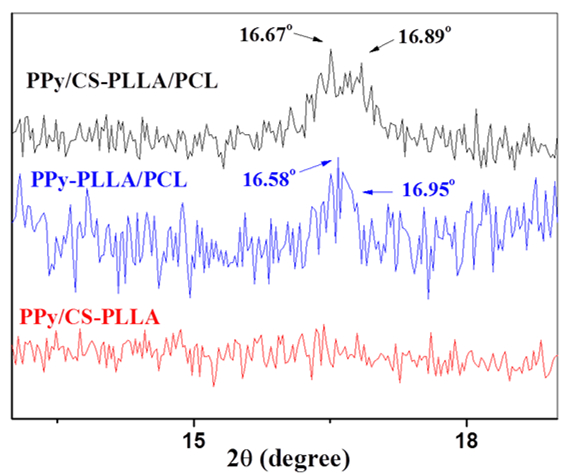


**3. Weight change of fiber-films in the immersion**

To research effect of CS on degradation of PLLA, an experiment was performed, and the residue weight of PPy/CS-PLLA/PCL or PPy-PLLA/PCL fiber-films were measured after they were immersed in normal saline (5 mL, pH = 6.89) for 1, 2, 4, 8 and 16 days. The original weights of two samples were both 0.054 g. Before weighted, the fiber-films were dried in drying oven for 48h.

As shown in Figure S4, the weights of two samples decreased with the time. However, the weight of PPy/CS-PLLA/PCL fiber-films was larger than that of PPy-PLLA/PCL fiber-films in 16 days. At the 2^nd^ day, the weight of PPy-PLLA/PCL fiber-films was significantly less than that of PPy/CS-PLLA/PCL fiber-films. At the 16^th^ day, the weight of PPy-PLLA/PCL fiber-films decreased to about 0.01 g, while the weight of PPy-PLLA/PCL fiber-films was about 0.02 g. These results indicate that the addition of CS decreased the degradation rate of PLLA to some extent, because the dedoped CS could balance the pH value of solution and reduce the cleavage of ester bonds caused by hydrogen ions.

**FIGURE S4.** The weight of PPy/CS- PLLA/PCL and PPy-PLLA/PCL fiber-films immersed in normal saline for different time. The asterisk * indicates significant differences (P < 0.05) between two corresponding groups.


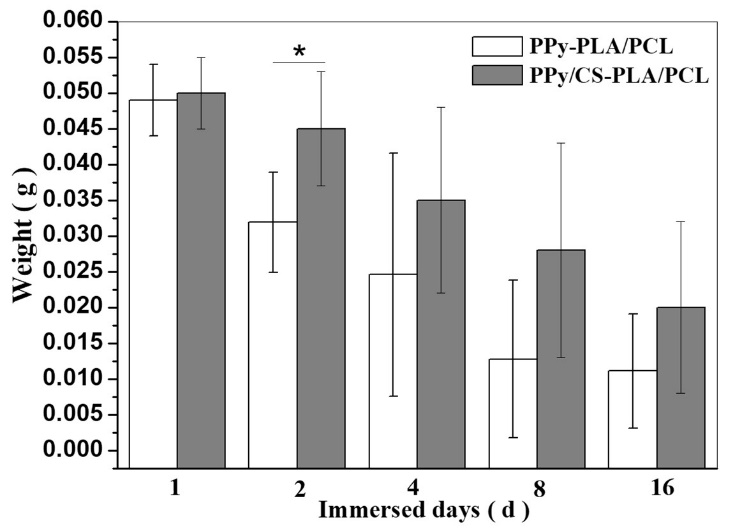


1. **Cell-compatibility of two fiber-films**

The MTT results of PC12 cells (FIGURE S5) show that the cyto-viability in PPy-PLLA/PCL groups was significantly lower than that of control groups at 3 days (P < 0.01), while the cyto-viability in CS/PPy-PLLA/PCL groups was significantly higher than that of control groups (P < 0.05). These results suggest that CS-doping in the composite fibers could promote the viability of cells. Furthermore, there was significant difference between the two treated groups of films with/without CS (P < 0.01), meaning that CS could enhance the cyto-compatibility of the prepared fiber-films.


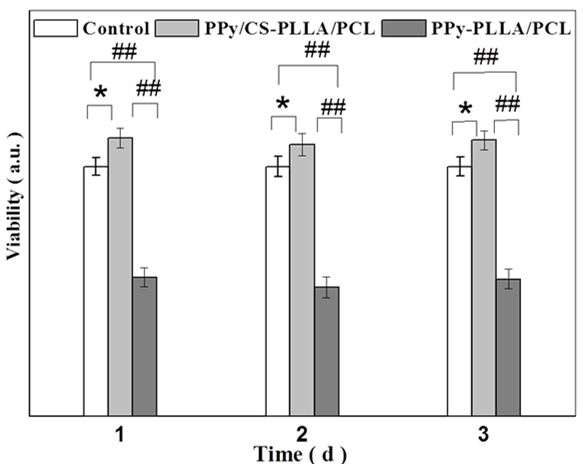

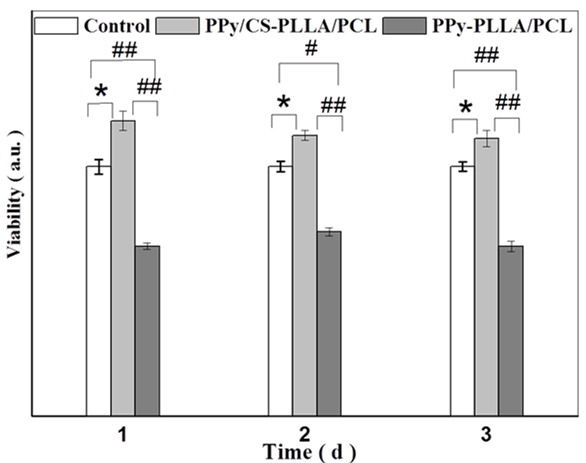


**a**

**b**

**FIGURE S5** MTT results of L929 cells (a) and PC12 cells (b) for the cyto-compatibility of CS/PPy-PLLA/PCL and PPy-PLLA/PCL fiber-films. The asterisk * indicates positive significant difference between two corresponding groups (P < 0.05); the mark # (P < 0.05) and ## (P < 0.01) indicate negative significant differences between two corresponding groups.

1. **Cell differentiation and neurite growth**

Figure S6 shows the images of PC12 cells cultured on CS/PPy-PLLA/PCL and PPy-PLLA/PCL fiber-films with or without ES for 1, 3 and 5 days. The differentiated PC12 cells and the extended neurites could be observed clearly. The orange arrowheads represent direction of fibers axis. The neurites in each group were almost aligned to grow, and neurite length from the differentiated cells was increased with the cultivation time. Compared with no stimulus of PPy-PLLA/PCL fiber-films (Figure S6a-c), there were in electrical stimulation groups (Figure S6d-f). Analogously, no ES groups for CS/PPy-PLLA/PCL fiber-films showed less and shorter neurites, compared with ES groups (shown in Figure S6g-i, S6j-l). In addition, the cells cultured on CS/PPy-PLLA/PCL fiber-films outgrew more and longer neurites (Figure S6g-l), compared with those on PPy-PLLA/PCL fiber-films (Figure S6a-f). Figure S7 shows the morphological feature of a single cell cultured on CS/PPy-PLLA/PCL fiber-films with ES for 5 days. The length of axon in the image was more than 100 μm and parallel with the direction of fibers axis.


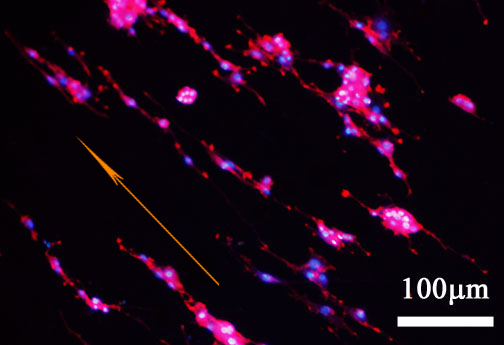

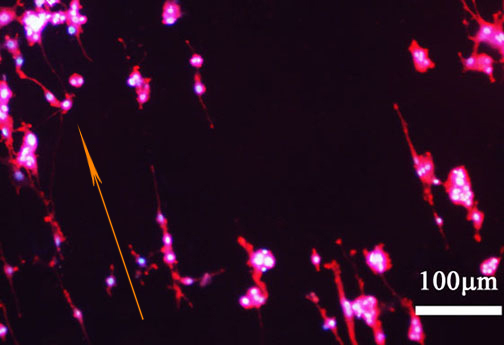

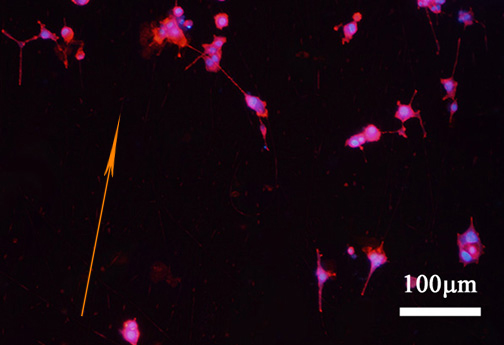

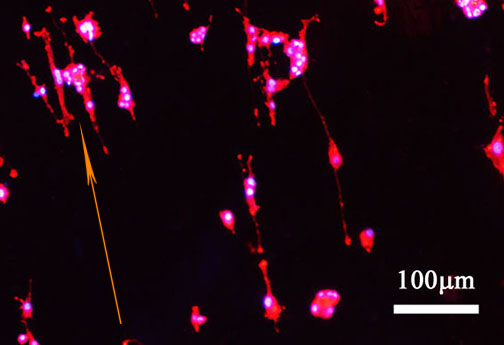

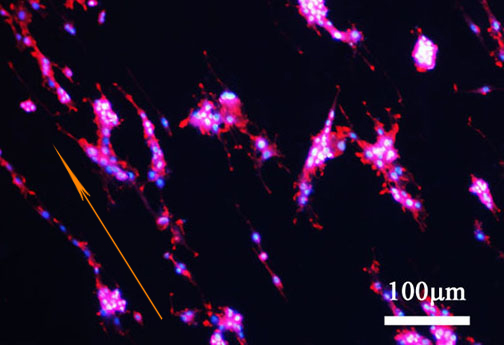

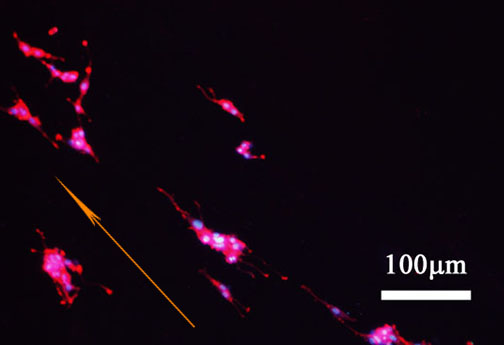

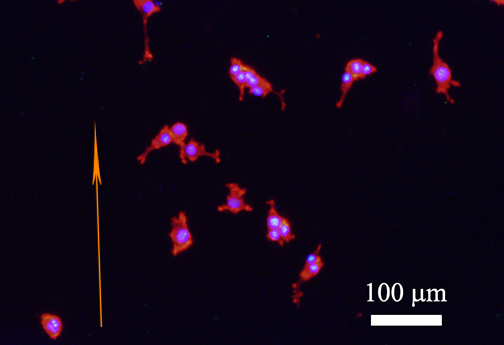

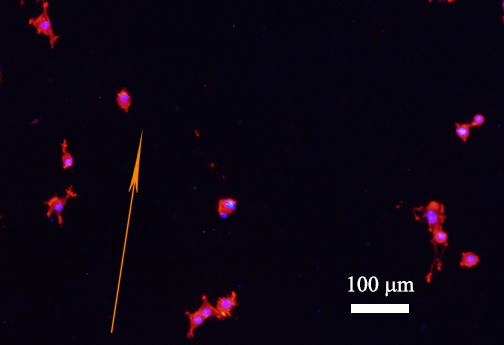

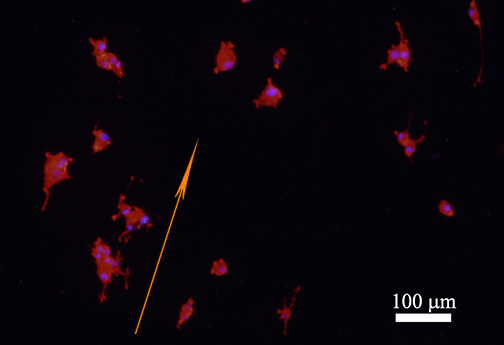


**FIGURE S6** Immunofluorescence (phalloidin/DAPI) micrograph of PC12 cells are shown above. The orange arrowheads represent direction of fibers axis. These cells were cultured on PPy-PLLA/PCL (a-f) or CS/PPy-PLLA/PCL (g-l) fiber-films for 1 (a, d, g, j), 3 (b, e, h, k) and 5 (c, f, i, l) days, with electrical stimulation (ES) of 100 mV for 2 hours every day (d-f, j-l) or without ES (a-c, g-i).

**e**

**h**

**i**

**g**

**j**

**k**

**l**

**d**

**f**


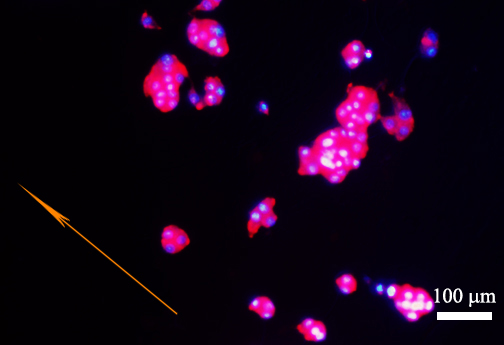

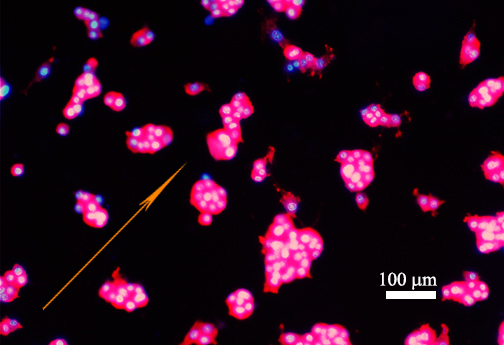

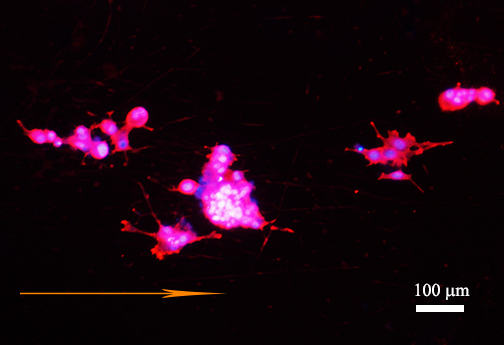


**FIGURE S7** Immunofluorescence (phalloidin/DAPI) micrograph of one PC12 cell.

**FIGURE S1** Schematic of electrostatic spinning.


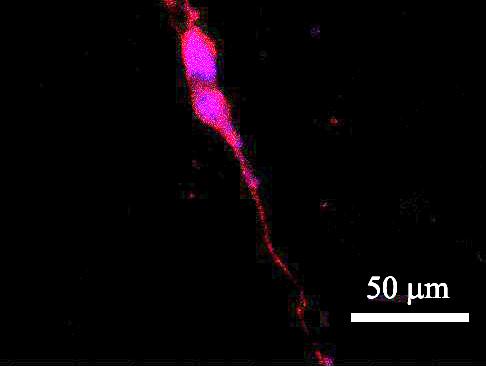


1. **Quantitative determination of MAP-2 concentrations**

Microtubule-associated protein-2 (MAP-2) serves to stabilize microtubules (MTs) growth by crosslinking MTs with intermediate filaments and other MTs. This protein implicates a role in determining and stabilizing dendritic shape during neuron development ^2^. ELISA kit of MAP-2 (ELISA LAB, Wuhan, China) was used to quantitatively determinate MAP-2 concentrations of PC12 cells cultured with various fiber-films and different time of electrical stimulation (ES) of 100 mV (1, 3 and 5 days, 2 h of ES every day).

The results in Figure S8 showed that, MAP-2 concentration from PPy/CS-PLLA/PCL group was larger than that from PPy-PLLA/PCL group, demonstrating that CS in fiber-films could support MAP-2 protein production, thus improving the differentiation of PC12 cells on fiber-films. To analyze effect of ES from fiber-films on the cells, PC12 cells of experimental groups were placed into cell plate with fiber-films and electrically stimulated for 1, 3 and 5 days, and 2 h per day, while the group without ES was used as control. The MAP-2 concentration of ES group in PPy/CS-PLLA/PCL film reached to ~ 633 pg·mL^-1^ at 5 d, higher than that of no ES groups (~ 602 pg·mL^-1^), indicating that ES could improve differentiation of PC12.

**FIGURE S8** MAP-2 concentration of PC12 cells in different culture conditions for 1, 3 and 5 days. The asterisk * (P < 0.05) and ** (P < 0.01) indicate positive significant differences between corresponding two groups.


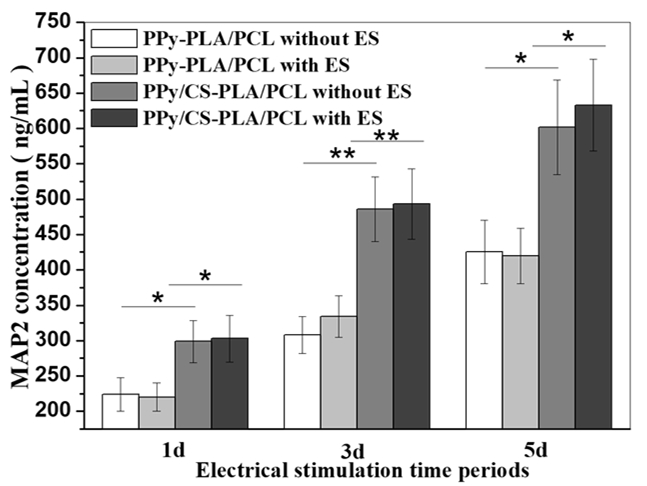


1. **References:**

1 Kayaci F, Umu OCO, Tekinay T. Antibacterial Electrospun Poly(lactic acid) (PLA) Nanofibrous Webs Incorporating Triclosan/Cyclodextrin Inclusion Complexes. J Agric Sci, **2013**; 61: 3901-3908.

2 Neve RL, Harris P, Kosik KS, et al. Identification of cDNA clones for the human microtubule-associated protein tau and chromosomal localization of the genes for tau and microtubule-associated protein 2. Brain Res, **1986**; 387: 271-280.
